# The Effect of a Multidisciplinary Lifestyle Intervention on Obesity Status, Body Composition, Physical Fitness, and Cardiometabolic Risk Markers in Children and Adolescents with Obesity

**DOI:** 10.3390/nu11010137

**Published:** 2019-01-10

**Authors:** Young-Gyun Seo, Hyunjung Lim, YoonMyung Kim, Young-Su Ju, Hye-Ja Lee, Han Byul Jang, Sang Ick Park, Kyung Hee Park

**Affiliations:** 1 Department of Family Medicine, Hallym University Sacred Heart Hospital, Anyang, Gyeonggi-do 14068, Korea; yg035@daum.net; 2 Department of Medical Nutrition, Kyung Hee University, Yongin, Gyeonggi-do 17104, Korea; hjlim@khu.ac.kr; 3 University College, Yonsei University International Campus, Incheon 21983, Korea; yoonkim@yonsei.ac.kr; 4 Department of Occupational and Environmental Medicine, Hallym University Sacred Heart Hospital, Anyang, Gyeonggi-do 14068, Korea; zorro@hallym.ac.kr; 5 Center for Biomedical Sciences, Korea National Institute of Health, Cheongju, Chungbuk 28159, Korea; hyejalee@korea.kr (H.-J.L.); greatstar@korea.kr (H.B.J.); parksi@nih.go.kr (S.I.P.)

**Keywords:** intervention, lifestyle, nutrition, exercise, body composition, cardiometabolic risk, severe obesity, childhood obesity, adolescents, pediatrics

## Abstract

This study aimed to develop a multidisciplinary lifestyle intervention program targeted at children and adolescents with moderate to severe obesity, and assess the additional effects of exercise intervention when compared to usual care. Overall, the 103 enrolled participants were ≥85th percentile of age and sex-specific body mass index (BMI). Participants were divided into groups that received 16 weeks of either usual care or exercise intervention. The BMI *z*-score of the overall completers decreased by about 0.05 after the 16-week intervention (*p* = 0.02). After the intervention, only the exercise group had a significantly lower BMI *z*-score than the baseline score by about 0.1 (*p* = 0.03), but no significant group by time interaction effects were observed. At the 16-week follow-up, significant group by time interaction effects were observed in percentage body fat (%BF) (β = −1.52, 95%CI = −2.58–−0.45), lean body mass (LM) (β = 1.20, 95%CI = 0.12–2.29), diastolic blood pressure (β = −5.24, 95%CI = −9.66–−0.83), high-sensitivity C-reactive protein (β = −1.67, 95%CI = −2.77–−1.01), and wall sit test score (β = 50.74, 95%CI = 32.30–69.18). We developed a moderate-intensity intervention program that can be sustained in the real-world setting and is practically applicable to both moderate and severe obesity. After interventions, the exercise group had lower %BF and cardiometabolic risk markers, and higher LM and leg muscle strength compared to the usual care group.

## 1. Introduction

Over the past few decades, the prevalence of obesity in children and adolescents has increased worldwide [1,2,3]. Children and adolescents with obesity are at high risk of transitioning to adult obesity [4]. Previous studies have found that obesity in childhood and adolescence increases the incidence of metabolic syndrome (MetS) and cardiovascular disease (CVD) such as hypertension (HTN), type 2 diabetes mellitus (T2DM), dyslipidemia (DL), and arteriosclerosis in adulthood [5,6], and also increases the risk of CVD in childhood and adolescence [7].

Particularly in childhood and adolescence, as the prevalence of obesity increases, the prevalence of severe obesity also increases in westernized countries [1,2,8]. In Korea, according to the statistical analysis of a sample of student health examination data in 2016, the obesity rate of elementary, middle, and high school students increased from 11.6% in 2007 to 16.5% in 2016, and the severe obesity rate has more than doubled in 10 years, from 0.8% in 2007 to 1.9% in 2016 [9]. When compared to obese children, children with severe obesity are at greater risk of adult obesity, early atherosclerosis, HTN, T2DM, DL, MetS, obstructive sleep apnea syndrome, fatty liver disease, and premature death [8]. Severely obese children and adolescents have a threefold increased risk of MetS compared to moderately obese children and adolescents [10,11]. An increasing gradation of obesity was associated with a higher risk of HTN, while severe obesity has a nearly threefold increased risk compared to moderate obesity in children and adolescents [12]. The Bogalusa Heart Study demonstrated that 39% of children with moderate obesity had at least two cardiovascular risk factors, while 59% of children with severe obesity had at least two cardiovascular risk factors [13].

A recent systematic review has shown that lifestyle-based weight loss interventions with 26 or more hours of intervention contact are likely to reduce excess weight in children and adolescents after six to 12 months [14]. Another recent review of studies on children and adolescents with morbid obesity showed positive weight loss results, indicating that younger children have a greater reduction in body mass index (BMI) *z*-score compared to adolescents. This emphasizes the importance of providing multidisciplinary interventions early in life and tailoring interventions to the needs of adolescents [15]. However, many of these previous intervention programs for obese children and adolescents were difficult to apply in real-world settings, such as home-visit models [16], high intensity intervention programs [17], or inpatient treatment [18,19]. Therefore, when the children and adolescents return to the real-world setting, they regain their weight [15,20]. Thus, we developed a moderate-intensity multidisciplinary lifestyle intervention program that is both sustainable in the real-world setting and practically applicable to both moderate and severe obesity. We also assessed the effectiveness of our intervention program on obesity status, body composition, physical fitness, and cardiometabolic risk markers.

## 2. Materials and Methods

### 2.1. Study Design and Participants

Overall, 103 participants aged between six and 16 years (63 boys and 40 girls), with a BMI ≥85th percentile of age and sex-specific BMI according to the 2007 Korean National Growth Charts [21], were recruited for the Intervention for Childhood and Adolescents Obesity via Activity and Nutrition (ICAAN) study through recruitment notices on the website, other social networking services, television (TV), newspapers, and flyers. Overweight was defined as a BMI ≥85th percentile for age and sex, while mild to moderate obesity was defined as a BMI ≥25 kg/m^2^ or ≥95th percentile for age and sex, while severe obesity was defined as a BMI ≥35 kg/m^2^ or ≥120% of the 95th percentile [21,22]. There are several studies that set BMI ≥97th percentile as the inclusion criteria for studies on severely obese children and adolescents [23,24]. Therefore, most of the participants in this study had moderate to severe obesity.

**Figure 1:**
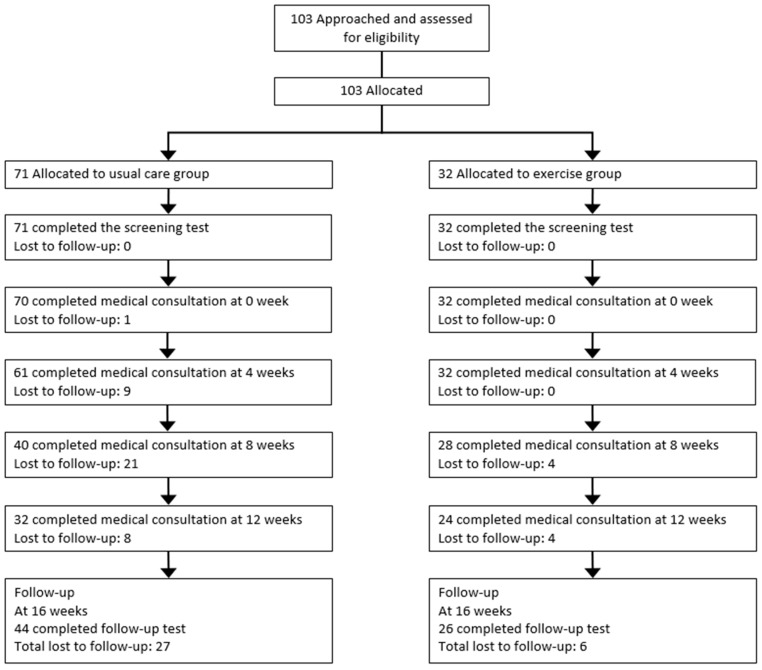
Participant flowchart.

**Figure 2:**
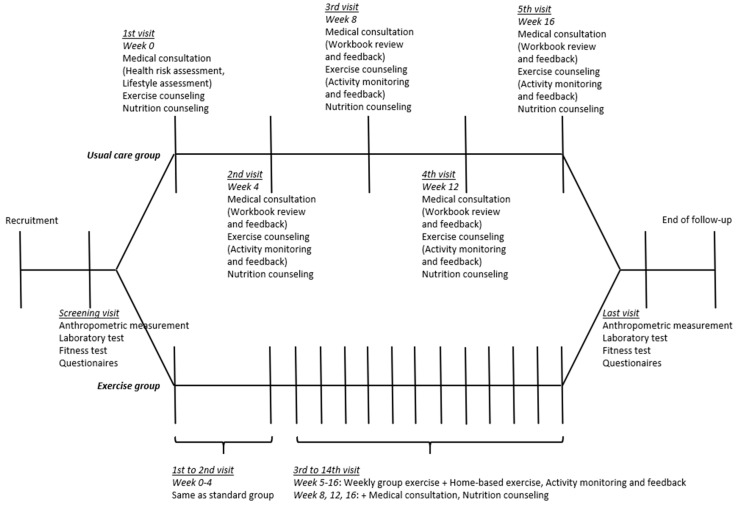
Intervention flowchart.

The ICAAN study was designed to test an intervention preventing excessive weight gain and to improve several health indices in children and adolescents with obesity in Korea. The design of the ICAAN study is a quasi-experimental intervention trial with two active treatment groups receiving a 16-week intervention program, which included 16 weeks of usual care (usual care group) or exercise (exercise group). This program is a multidisciplinary program that includes medical consultation and behavioral modification by doctors, nutritional counseling and education by clinical dietitians, exercise training and fitness test by exercise specialists, evaluation and assessment of the family environment by social workers, physical measurement and blood sampling by nurses, and lifestyle monitoring and feedback through smart bands by research coordinators. The flowcharts describe the strategy of recruiting the participants and administering the intervention (Figure 1 and Figure 2). We used a consecutive randomization procedure as a principle in assigning participants, but also considered geographic reasons and personal circumstances. A senior researcher who was not blinded to the research aims carried out the allocation.

This study was conducted according to the guidelines laid down in the Declaration of Helsinki, and all of the procedures involving human participants were approved by the Hallym University Sacred Heart Hospital’s Institutional Review Board (approval number: 2015-I134). Written informed consent was obtained from all of the participants and their parents or caregivers. This study was registered at cris.nih.go.kr (identifier: KCT0002111).

### 2.2. Intervention

#### 2.2.1. Usual Care Group

All of the ICAAN programs were conducted within the hospital. The conformity of the intervention was monitored by the Korea Centers for Disease Control and Prevention, and performed as planned during the follow-up period. Figure 2 shows the flowchart of the intervention. All of the participants received usual care. Usual care consisted of one-to-one medical consultation, workbook provision for goal setting and behavioral modification, exercise counseling, physical activity monitoring and feedback, and one-to-one nutritional counseling.

At the first visit, a doctor explained the results of the anthropometric measurements and laboratory tests, and assessed the health risks and lifestyle of all of the participants. The doctor conducted four-weekly one-to-one medical consultations during the intervention for goal setting, after reviewing the workbooks. The contents of the workbook are shown in Table 1.

In addition to medical consultations, all of the participants received exercise counseling from the doctor, focusing on an increase in the amount of physical activity and reduction in inactive time. To ensure and promote participants’ daily physical activity levels, all of the participants were asked to wear a physical activity tracker (iBODY24 Planner +, Greencomm, Korea) throughout the intervention period. All of the participants were instructed to walk more than 8000 steps per day, and a text message was sent once a week to encourage daily physical activity.

One-to-one nutritional counseling, according to the Nutritional Care Process (NCP) protocol, was conducted every four weeks by a clinical dietitian for all of the participants. The NCP contains four distinct but interrelated steps: nutritional assessment, diagnosis, intervention, and monitoring/evaluation; and it enabled systematic and efficient nutritional counseling. The contents of the nutritional intervention comprised five sessions (Table 2), and each session was conducted with customized nutritional counseling for 25 min.

#### 2.2.2. Exercise Group

The participants in the exercise group also received the usual care during the intervention period. In addition, they were instructed to participate in a 12-week exercise program from the fifth week of the intervention. The exercise program required participants to exercise three days/week for 60 min/session (one group exercise session and two home-based exercise sessions) at 60% to 90% of the maximal heart rate (HR). Our group exercise program is a circuit training using body weight, consisting of six different exercises (Table 3). Each exercise was performed for one minute, with 30–40 s rest, and it took approximately nine to 10 min to complete one bout of the entire workout (named an ICAAN Exercise). During group exercise, participants wore a HR monitor (Polar H7 Bluetooth HR Sensor, Polar, Finland) to ensure an appropriate level of exercise intensity. The home-based exercise was a review process of the group exercise. The goal was to perform the following more than twice a week at home: 30 min of aerobic exercise such as running or cycling as well as an ICAAN Exercise for 30 min. In the home-based exercise, a physical activity tracker was used for monitoring, and the daily exercise journal completed by the exercise group was reviewed weekly for feedback.

### 2.3. Questionnaire

The questionnaires completed by the children and adolescents included dietary habits, physical activity, drinking, smoking, sleeping time, screen time (TV/computer use), inactive time, and mental health (depression, stress, etc.). The questionnaire items completed by the parents or caregivers included education, monthly household income, past medical history, marital status, and the child’s birth-related variables (birth weight, type of feeding, etc.). We used the Children’s Depression Inventory [25,26,27], and the Global Physical Activity Questionnaire [28], all of which are valid questionnaires.

### 2.4. Dietary Assessment

Dietary intakes were collected using three-day food records (two weekdays and one weekend day). The participants’ nutrient intakes were assessed using a computer-aided nutritional analysis program (CAN Pro, Web version 5.0, The Korean Nutrition Society, Seoul, Korea, 2015).

### 2.5. Anthropometric Measurements and Body Composition

Body weight and body composition were measured by Bioelectrical Impedance Analysis (InBody 720 Body Composition Analyzer, BioSpace Co., Ltd., Seoul, Korea) after a 10-hour fast and voiding, with the participant barefoot, wearing indoor and lightweight clothing. Height was measured by a stadiometer (DS-103, DongSahn Jenix, Seoul, Korea), with the participant barefoot. Weight and height were measured to the nearest 0.1 kg and 0.1 cm, respectively. BMI values (weight in kilograms divided by height in meters squared) were converted into percentiles and *z*-scores were based on the age and sex-specific BMI of the 2007 Korean National Growth Charts [21]. Waist circumference (WC) was measured at the midpoint between the last rib and the top of the iliac crest to the nearest 0.1 cm, using a non-elastic tape measure. The measurements of these variables were also carried out in parents. To assess body composition, an additional whole-body dual-energy X-ray absorptiometry (DXA) (Lunar Prodigy Advance with pediatric software version enCore 14.0, GE Medical Systems Lunar, Madison, WI, USA) scanner performed a series of transverse one-centimeter scans starting at the participant’s head and progressing toward the feet after a 10-hour fast and voiding. DXA assessments were made by a certified radiology technologist. Finally, we used the values measured in the DXA for body composition analysis.

### 2.6. Blood Pressure and Blood Samples

The mean value of the blood pressure (BP) of participants was measured twice in the right arm in the seated position using an oscillometric device (DINAMAP ProCare 200, GE Medical Systems, Milwaukee, WI, USA). Venous blood samples were obtained after 12 hours of fasting to determine the fasting plasma glucose (FPG), fasting plasma insulin, high-density lipoprotein cholesterol (HDL-C), low-density lipoprotein cholesterol (LDL-C), triglyceride (TG), aspartate aminotransferase (AST), alanine aminotransferase (ALT), gamma-glutamyl transferase (GGT), and high-sensitivity C-reactive protein (CRP). FPG was measured using ultraviolet assay with hexokinase (Cobas 8000 C702, Roche, Mannheim, Germany). Insulin was measured using electrochemiluminescence immunoassay (Cobas 8000 e802, Roche, Mannheim, Germany). HDL-C and LDL-C were measured using a homogeneous enzymatic colorimetric test (Cobas 8000 C702, Roche, Mannheim, Germany). TG, AST, ALT, and GGT were measured using enzymatic assay (Cobas 8000 C702, Roche, Mannheim, Germany). CRP was measured using turbidimetric immunoassay (Cobas 8000 C702, Roche, Mannheim, Germany). The homeostasis model assessment for insulin resistance (HOMA-IR) was used to determine insulin sensitivity, and was calculated using the following formula: (FPG Level (in milligrams per deciliter) × Fasting Plasma Insulin Level (in microunits per milliliter))/405.

### 2.7. Fitness Test

Cardiorespiratory fitness and muscular strength tests were performed by trained professional personnel using the step test, arm (bicep) curl test, and wall sit test. In the step test, to measure cardiorespiratory fitness, participants climbed up and down a 30-cm height step box for three minutes to match the metronome speed set at 96 beats/min (24 steps/min). Post-exercise HR was measured for one minute after the end of the test. The arm curl test measured the muscular strength of the upper extremities. We measured the number of times the full extension and curl was performed for 30 s with a two kg (female) or four kg (male) dumbbell while sitting in a chair. The wall sit test was used to measure the muscular strength of the lower extremities. We measured the time taken to maintain posture by leaning against the wall as if sitting on a desk with legs open to shoulder width leg.

### 2.8. Procedures

The primary outcome of the study was the BMI *z*-score, standardized by the use of age and sex-specific BMI from the 2007 Korean National Growth Charts [21], and percentage of the 95th percentile of age and sex-specific BMI (%BMI_p95th_). Secondary outcomes included (1) body composition variables (WC, body fat (BF), and lean body mass (LM)); (2) cardiometabolic risk markers (BP, FPG, insulin, HOMA-IR, HDL-C, LDL-C, TG, AST, ALT, GGT, and CRP); (3) nutrition (total energy intake); and (4) cardiorespiratory fitness and muscular strength. All of the questionnaires and tests were completed at baseline and at 16 weeks.

### 2.9. Sample Size Estimation

Sample size estimation at 80% power with a two-sided significance level set at 0.05 showed that a minimum of 34 participants, 17 in each group, were required to observe significant differences in the BMI *z*-score changes between groups. We used sample size estimation for a two-sample means test under the null hypothesis indicating that the means of the two groups are equal. We assumed that the response within each group is normally distributed with a standard deviation of 0.1 and a true difference in BMI *z*-score changes between groups of 0.1.

### 2.10. Statistical Analysis

The Shapiro–Wilk test was used to evaluate the normality of the data. The analysis was conducted by transforming the data to a logarithmic scale to achieve a bell-shaped (approximately normal) distribution on the required variables. We compared the baseline characteristics of the two treatment groups using two-tailed *t* tests for independent samples (after checking for normal distributions of continuous variables). In addition, we used the Pearson’s chi-squared test or Fisher’s exact test for categorical variables. The paired *t* test was used to compare the pre-intervention and post-intervention outcomes (baseline and at 16 weeks). Mixed effects linear regression models for repeated measures were used to analyze between-group differences in primary and secondary outcomes over time. Intercept was used for the mixed model random effects at the individual level.

All of the statistical analyses were conducted using Stata/MP, version 14.0 (StataCorp, College Station, TX, USA). All of the statistical tests were two-sided, and statistical significance was determined at a *p*-value < 0.05.

## 3. Results

### 3.1. Baseline Characteristics

Of the 103 participants, 38.8% were girls, 34.0% had severe obesity, 80.6% had a BMI ≥97th percentile for age and sex, and the mean age of the participants was 12.56 ± 1.96 years. There were no significant differences in the proportion of severe obesity (31.0% versus 40.6%, *p* = 0.34) and the proportion of BMI ≥97th percentile for age and sex (81.7% versus 78.1%, *p* = 0.67) between usual care and exercise groups at baseline. After the 16-week intervention period, 70 participants were followed up, and the follow-up rate was 68.0%. The reasons for the loss to follow-up were: parents or family-related problem (*n* = 4, 12.1%), child-related problem (*n* = 6, 18.2%), lack of willingness (*n* = 3, 9.1%), disconnection (*n* = 5, 15.2%), and no data (*n* = 15, 45.5%). There were no significant differences in the main outcomes between the followed-up and dropped-out groups at baseline, except for HDL levels (48.23 versus 43.55, *p* = 0.02).

We defined participants who have data at both baseline and 16-week follow-up as completers, and performed analysis on all of the participants (*n* = 103) and completers (*n* = 70) separately, using baseline data. Table 4 shows the anthropometric measurements and the secondary outcome variables for all of the participants and completers. There were no significant differences between the usual care and exercise groups at baseline.

### 3.2. Changes in Primary Outcomes

The overall completers’ baseline and 16-week BMI *z*-scores were 2.32 and 2.27, respectively; there was a statistically significant decrease after the intervention (*p* = 0.02). After the intervention, only the exercise group had significantly lower BMI z-scores (2.24 ± 0.56 versus 2.32 ± 0.52, *p* = 0.03) and %BMI_p95th_ (114.10 ± 1.13% versus 116.02 ± 1.12%, *p* = 0.04) than at baseline (Table 5). However, after adjustment for confounding variables including sex, age, parental obesity, parental education, monthly household income, marital status, and sleep time, by mixed effects linear regression, there were no statistically significant differences in the BMI z-score and %BMI_p95th_ between the two intervention groups (β −0.02, 95%CI −0.13 to 0.10; β −1.00, 95%CI −1.03 to 1.02, respectively) after the 16-week follow-up (Table 5).

### 3.3. Changes in Secondary Outcomes: Body Composition, Cardiometabolic Risk Markers, Nutrition, and Cardiorespiratory Fitness, and Muscular Strength

After the intervention, both groups had higher LM (usual care group: 41.26 ± 10.96 kg versus 40.18 ± 10.98 kg, *p* < 0.001; exercise group: 44.55 ± 6.04 kg versus 42.85 ± 6.41 kg, *p* < 0.001) and lower total energy intake (usual care group: 2022.5 ± 521.6 kcal versus 2367.2 ± 424.9 kcal, *p* = 0.001; exercise group: 1965.4 ± 409.0 kcal versus 2509.8 ± 559.9 kcal, *p* < 0.001) than at baseline (Table 6). In the usual care group, BF (*p* = 0.01) at the 16-week follow-up was higher than at baseline (Table 6). In the exercise group, the percentage of body fat (%BF) (*p* < 0.001), diastolic blood pressure (DBP) (*p* = 0.03), ALT (*p* = 0.002), GGT (*p* = 0.01), and CRP (*p* = 0.02) at the 16-week follow-up were lower than at baseline, while the arm curl test score (*p* = 0.02) and wall sit test score (*p* < 0.001) at the 16-week follow-up were higher than at baseline (Table 6).

The mixed effects linear regression analysis adjusted for age and sex showed significant differences in BF (β −1.25, 95%CI −2.22 to −0.28), %BF (β −1.31, 95%CI −2.07 to −0.54), LM (β 0.76, 95%CI 0.02 to 1.50), GGT (β −1.15, 95%CI −1.27 to −1.04), CRP (β −1.61, 95%CI −2.33 to −1.11), and wall sit test score (β 34.26, 95%CI 19.65 to 48.88) between the two intervention groups after the 16-week follow-up. After adding potential confounders including parental obesity, parental education, monthly household income, marital status, and sleep time to the multivariable regression model, significant group by time interaction effects were still observed in %BF (β −1.52, 95%CI −2.58 to −0.45), LM (β 1.20, 95%CI 0.12 to 2.29), DBP (β −5.24, 95%CI −9.66 to −0.83), CRP (β −1.67, 95%CI −2.77 to −1.01), and wall sit test scores (β 50.74, 95%CI 32.30 to 69.18).

## 4. Discussion

In this study, two types of lifestyle interventions for obese children and adolescents were compared. In the exercise group, the BMI *z*-score significantly decreased by about 0.1 after 16 weeks of intervention compared with the score at baseline, but there was no significant difference in the BMI *z*-scores between the usual care and exercise groups. This study also demonstrated that in children and adolescents with obesity, the intervention achieved positive effects on body composition, physical fitness, and cardiometabolic risk markers. At the 16-week follow-up, significant improvement over the intervention period was found in the exercise group compared with the usual care group with %BF, LM, DBP, CRP, and wall sit test score.

A recent systematic review has shown that lifestyle-based weight management interventions, above a threshold of 26 estimated contact hours, were generally effective in reducing excess weight in children and adolescents after six to 12 months. This was typically seen with absolute BMI z-score reductions of 0.2 or more, compared with little or no reduction in the control groups [14]. However, interventions with fewer than the estimated 26 h of contact, over three to 24 months, showed statistically significant benefits at the six to 12-month follow-up, and the standardized effect sizes were usually small, generally reflecting absolute BMI *z*-score reductions of 0.1 or less in the intervention groups [14]. Moreover, an intervention study (which included participants who were overweight, had obesity, or severe obesity) suggested that an intensive behavioral weight loss intervention that has demonstrated efficacy in decreasing BMI *z*-scores may have incrementally smaller effects in children as weight classification increases [29]. Therefore, considering that this study is a moderate-intensity intervention applicable to both moderate and severe obesity, the effect size was not significantly different from these previous studies. Furthermore, it is difficult to expect a large difference between the two groups in this study, because the usual care group, which is an active comparator, received monthly medical counseling, exercise counseling, and nutritional counseling (NCP protocol), unlike the controls in other studies.

For the body composition, we observed significant improvement not only of %BF, but also of LM in the exercise group compared to the usual care group. Previous randomized controlled trials for inpatient or family-based treatment of children and adolescents with severe obesity has shown group by time interaction effects for %BF [18,23]. However, in some previous studies, fat-free mass (FFM) rather decreased after interventions, and a reduction in FFM was associated with a decrease in metabolic rate. This appears to be a major factor in later weight regain in obese children [19,30]. These previous intervention programs included moderate calorie restriction; it is well-known that hypocaloric diets result in a decreased resting metabolic rate by the reduction in FFM [31,32]. In addition to the physiologic increase in LM due to growth, balanced diet and personalized nutritional education rather than energy restriction seem to have contributed to the positive effect of body composition in this study. Therefore, at least the participants in this study may be expected to have no later weight regain.

In this study, we observed improvements in DBP and CRP. Several studies have reported positive effects on BP after interventions [14,23]. However, cardiometabolic outcomes were sparsely reported in trials of less intensive interventions, and were generally not associated with improvements in BP, lipid levels, or insulin or glucose levels. In a recent review of studies on children and adolescents with morbid obesity, cardiovascular risk parameters were only investigated in three of the included studies; the results demonstrated that children achieved positive effects on cardiometabolic risk markers (LDL, TG, and insulin sensitivity) [15]. Considering the relatively short duration and moderate intensity of this intervention, there might be a limitation in the improvement of all of the cardiometabolic indices.

Recent studies have shown that grip strength and other muscle strengths as well as various strength trainings have positive effects on cardiometabolic outcomes [33,34,35,36]. Therefore, in this study, improvement in the strength of the lower extremities (measured by the wall sit test in the exercise group after the intervention) could be expected to have a positive effect on cardiometabolic outcomes, in addition to the effect of weight control in obese children and adolescents.

In this study, the follow-up rate was 68% after the 16-week intervention period. While some previous studies had follow-up rates of more than 70% during the intervention periods of three to six months [37,38,39], others had follow-up rates of less than 70% [40,41]. In the intensive intervention study or the study on severe obesity, the follow-up rate tended to be low [42,43]. Overall, the follow-up rates varied from 60% to 85% during intervention periods of three to six months in previous intervention studies on obesity among children and adolescents [37,38,39,40,41,42,43]. Therefore, this study seems to be feasible because it showed a follow-up rate close to 70%, even though it included severe obesity, and that moderate intensity intervention was performed.

This study had some limitations. In spite of the consecutive randomization procedure, we could not completely allocate the participants in both groups randomly for geographical reasons and personal circumstances. However, there were no significant differences in the main outcomes between the usual care and exercise groups at baseline. Another limitation is the short duration of the follow-up. While it is important to evaluate the long-term efficacy of this intervention, our primary objective was to ensure the feasibility and acceptability of our intervention program before launching a larger and longer trial. In addition, the small sample size was also a limitation. As a final limitation, we did not assess the multi/interdisciplinarity of the team. However, during the program, there was no problem with the integration or harmonization of our team, subjectively.

The strength of this study is that the protocol was intended to be easily applied in real-world settings. Many of the previous best practice interventions for obese children and adolescents were difficult to apply in real-world settings; hence, the weights were regained after returning to the real-world settings [15,20]. Therefore, we developed an intervention program that can be easily performed and can be continued on its own after the intervention, by reflecting the real-world setting. As a result, positive effects were observed on body composition, cardiovascular risk markers, and physical fitness in the exercise group (who were trained through repeated circuit training that can be easily performed at home), compared to the usual care group. In other words, a better effect was obtained by adding a practically applicable exercise program to the usual obesity care. Another strength of this study is the expectation that weight regain can be prevented by continuing with this exercise program.

## 5. Conclusions

We have developed a moderate-intensity multidisciplinary lifestyle intervention program that can be sustainable in the real-world setting and practically applicable to both moderate and severe obesity. After a 16-week multidisciplinary lifestyle intervention, the exercise group had a lower %BF and cardiometabolic risk markers, with higher LM and leg muscle strength, compared to the usual care group.

It is expected that the physical fitness and cardiometabolic risk markers as well as the body composition in obese children and adolescents can be improved through education and practicing circuit training exercises, such as the ICAAN Exercise, which obese children and adolescents can easily follow. This should be done in conjunction with nutrition and psychological counseling in clinical practice. Therefore, a long-term intervention is necessary to confirm the effectiveness of this program on moderate to severe obese children and adolescents.

## Figures and Tables

**Table 1:** The contents of the workbook.

Visit	Title	Contents
Screening	Know my body	Baseline tests
1st visit	Learn my body 1	One-to-one medical consultation/Goal setting.
	Manage my body 1–1	Why should I adjust my weight?
	Manage my body 1–2	Let’s take a look at my lifestyle.
	Manage my body 1–3	What makes this process so challenging?
2nd visit	Learn my body 2	What changes in my body?
	Manage my body 2–1	What is the obstacle to my weight control?
	Manage my body 2–2	What is the lifestyle of our family?
	Manage my body 2–3	My appearance that I think, and my appearance that others think.
3rd visit	Learn my body 3	What changes in my body?
	Manage my body 3–1	Eating only a small amount cannot control my weight.
	Manage my body 3–2	Keep my daily life healthy.
	Manage my body 3–3	Be confident.
4th visit	Learn my body 4	What changes in my body?
	Manage my body 4–1	What makes this process so challenging?
	Manage my body 4–2	How much have I changed?
	Manage my body 4–3	Weight control is not done at once.
5th visit	Keep my body	2nd tests/One-to-one medical consultation.

**Table 2:** Monthly key messages and action plan for nutritional counseling.

Session	Contents
1	My meal planning
2	My meal portion
3	Choose healthy snack
4	Cook with fruits and vegetables
5	Maintain my healthy diet

**Table 3:** Weekly schedule and protocol for Intervention for Childhood and Adolescents Obesity via Activity and Nutrition (ICAAN) Exercise.

Week	ICAAN Exercise Protocol
1/7	Butt Kicker → Diagonal Chop → Plank Jack → Skater Jump → Squat → Plank
2/8	Halo Slam → Power Skip → Rotational Chop → Running Mountain Climber → Lunge → Plank
3/9	Alternating Fast Feet → In and Out Squat → Side to Side Mountain Climber → Skier Swing → Wall Sit → Plank
4/10	Jumping Jack → Long Jump to Backward Hop → Punch → Slam → Wall Squat → Plank
5/11	High Knee Run → Mountain Climber → Speed Walkout → Box Squat Jump → Buffy → Plank
6/12	Squat → Lunge → Side to Side Mountain Climber → Jumping Jack → Buffy → Plank

**Table 4:** Baseline demographic characteristics, anthropometric measurements, laboratory test results, lifestyle measurements, and fitness test results of all participants and completers.

Characteristic	All Participants			Completers		
	Usual Care Group (*n* = 71)	Exercise Group (*n* = 32)	*p* Value	Usual Care Group (*n* = 44)	Exercise Group (*n* = 26)	*p* Value
**Age, year**	12.39 ± 2.07	12.92 ± 1.69	0.21	12.09 ± 2.20	12.80 ± 1.72	0.16
**Sex, No. (%)**			0.85			0.88
Male	43 (60.56)	20 (62.50)		28 (63.64)	17 (65.38)	
Female	28 (39.44)	12 (37.50)		16 (36.36)	9 (34.62)	
**Parental obesity, No. (%)** **(*n* = 57/25)/(*n* = 37/22) ^a^**			0.62			0.73
None	14 (24.14)	5 (19.23)		7 (18.92)	3 (13.64)	
Either	44 (75.86)	21 (80.77)		30 (81.08)	19 (86.36)	
**Parental CVD history, No. (%)** **(*n* = 55/24)/(*n* = 35/19) ^a^**			0.37			0.51
None	29 (52.73)	10 (41.67)		18 (51.43)	8 (42.11)	
Either	26 (47.27)	14 (58.33)		17 (48.57)	11 (57.89)	
**Parental education, No. (%)** **(*n* = 57/27)/(*n* = 38/23) ^a^**			0.63			0.81
<College (both)	18 (31.03)	7 (25.93)		11 (28.95)	6 (26.09)	
≥College (either)	40 (68.97)	20 (74.07)		27 (71.05)	17 (73.91)	
**Monthly household income, No. (%)** **(*n* = 57/26)/(*n* = 36/21) ^a^**			0.93			0.75
<3 million KRW	8 (14.04)	4 (15.38)		5 (13.89)	2 (9.52)	
3–5 million KRW	20 (35.09)	8 (30.77)		14 (38.89)	7 (33.33)	
≥5 million KRW	29 (50.88)	14 (53.85)		17 (47.22)	12 (57.14)	
**Marital status, No. (%)** **(*n* = 60/27)/(*n* = 39/23) ^a^**			0.17			0.35
Living together with parents	55 (91.67)	22 (81.48)		37 (94.87)	20 (86.96)	
Other situation	5 (8.33)	5 (18.52)		2 (5.13)	3 (13.04)	
**Birth weight, kg** **(*n* = 58/28)/(*n* = 39/23) ^a^**	3.32 ± 0.43	3.33 ± 0.61	0.96	3.39 ± 0.46	3.34 ± 0.64	0.76
**Body weight, kg**	73.17 ± 17.91	78.05 ± 13.38	0.17	72.16 ± 19.88	77.43 ± 11.34	0.22
**BMI, kg/m^2^**	29.56 ± 4.23	30.33 ± 4.06	0.39	29.43 ± 4.81	30.06 ± 3.40	0.56
**BMI *z*-score**	2.32 ± 0.45	2.34 ± 0.54	0.87	2.32 ± 0.48	2.32 ± 0.52	0.98
%BMI_p95th_, % *	115.95 ± 1.11	116.63 ± 1.14	0.81	116.22 ± 1.13	116.02 ± 1.12	0.95
**Waist circumference, cm**	93.50 ± 11.38	97.50 ± 9.58	0.09	93.43 ± 11.79	97.61 ± 8.59	0.12
**Body fat, kg**	30.38 ± 8.17	32.48 ± 7.65	0.22	29.75 ± 9.40	32.33 ± 6.38	0.22
**Body fat, %**	41.64 ± 3.77	41.53 ± 4.42	0.90	41.26 ± 4.25	41.77 ± 4.23	0.63
**Lean mass, kg**	40.57 ± 10.17	43.28 ± 6.89	0.17	40.18 ± 10.98	42.85 ± 6.41	0.26
**SBP, mmHg**	124.33 ± 14.71	126.27 ± 14.85	0.54	123.24 ± 15.69	125.92 ± 14.99	0.49
**DBP, mmHg**	66.87 ± 7.38	65.70 ± 6.72	0.45	66.67 ± 7.64	65.60 ± 6.72	0.55
**Laboratory test results**						
HOMA-IR *	4.33 ± 1.73	4.99 ± 2.01	0.27	4.17 ± 1.84	5.28 ± 2.09	0.15
HDL-C, mg/dL	46.44 ± 8.98	47.38 ± 10.60	0.64	47.45 ± 9.51	49.54 ± 10.42	0.40
LDL-C, mg/dL	114.24 ± 24.74	111.78 ± 26.35	0.65	113.59 ± 22.62	110.15 ± 28.04	0.58
TG, mg/dL *	102.82 ± 1.50	111.32 ± 1.63	0.39	98.27 ± 1.50	108.92 ± 1.63	0.34
AST, U/L *	22.76 ± 1.53	25.74 ± 1.86	0.24	23.30 ± 1.53	26.23 ± 1.90	0.36
ALT, U/L *	24.68 ± 2.13	29.90 ± 2.55	0.27	25.30 ± 2.22	31.56 ± 2.61	0.30
GGT, U/L *	20.42 ± 1.70	23.26 ± 1.87	0.28	20.23 ± 1.78	23.34 ± 1.96	0.35
CRP, mg/L *	1.49 ± 2.79	1.73 ± 2.64	0.50	1.44 ± 3.01	1.87 ± 2.76	0.32
**Nutrition**						
Total energy intake, kcal (*n* = 69/32) *	2362.5 ± 578.7	2548.0 ± 551.3	0.13	2368.6 ± 429.3	2509.8 ± 559.9	0.24
**Activity level**						
Sleep time, hour (*n* = 68/28)/(*n* = 44/22) ^a^	8.38 ± 1.21	8.54 ± 1.54	0.60	8.47 ± 1.22	8.47 ± 1.43	0.99
Inactivity time, hour (*n* = 62/28)/(*n* = 42/22) ^a^	2.76 ± 1.78	3.79 ± 3.19	0.05	2.77 ± 1.80	3.62 ± 2.89	0.15
**Cardiorespiratory fitness and muscular strength**						
Step test, post exam HR (*n* = 61/28)/(*n* = 38/22) ^a^	127.51 ± 21.85	128.46 ± 20.88	0.85	127.42 ± 22.14	123.95 ± 19.41	0.54
Arm curl test, frequency (*n* = 61/28)/(*n* = 38/22) ^a^	16.03 ± 6.36	17.00 ± 5.66	0.49	15.92 ± 7.13	16.86 ± 5.64	0.60
Wall sit test, sec (*n* = 61/28)/(*n* = 38/22) ^a^	43.11 ± 26.68	36.58 ± 23.70	0.27	47.11 ± 28.91	36.27 ± 23.19	0.14

Abbreviations: CVD, cardiovascular disease; KRW, Korean Republic Won; BMI, body mass index; SBP, systolic blood pressure; DBP, diastolic blood pressure; HOMA-IR, homeostasis model assessment for insulin resistance; HDL-C, high-density lipoprotein cholesterol; LDL-C, low-density lipoprotein cholesterol; TG, triglyceride; AST, aspartate aminotransferase; ALT, alanine aminotransferase; GGT, gamma-glutamyl transferase; CRP, high-sensitivity C-reactive protein; HR, heart rate. HOMA-IR = (fasting plasma glucose level (in milligrams per deciliter) × fasting plasma insulin level (in microunits per milliliter))/405. Data are presented as mean ± standard deviation for continuous variables (*t* test) and number (%) for categorical variables (χ^2^ test or Fisher’s exact test). Percentages have been rounded and may not total 100. * Geometric mean ± standard deviation. ^a^ Completers.

**Table 5:** Changes in primary outcomes of usual care and exercise groups.

Outcome Measure	Usual Care Group (*n* = 44)	Exercise Group (*n* = 26)	β (95%CI) ^b^	β (95%CI) ^c^
**BMI *z*-score**				
Baseline	2.32 ± 0.48	2.32 ± 0.52		
16-wk follow-up	2.29 ± 0.53	2.24 ± 0.56	−0.04 (−0.12 to 0.03)	−0.02 (−0.13 to 0.10)
*p*-value ^a^	0.19	0.03		
%BMI_p95th_ *				
Baseline	116.22 ± 1.13	116.02 ± 1.12		
16-wk follow-up	115.37 ± 1.14	114.10 ± 1.13	−1.01 (−1.03 to1.01)	−1.00 (−1.03 to 1.02)
*p*-value ^a^	0.16	0.04		

Abbreviations: CI, confidence interval; BMI, body mass index; wk, week. Data were expressed as mean ± standard deviation unless otherwise indicated. * Geometric mean ± standard deviation; ^a^ paired *t*-test between baseline data and 16-wk follow-up data. ^b^ group × time interaction effects adjusted for age and sex in the mixed effects linear regression models (random intercept: individual). ^c^ group × time interaction effects adjusted for age, sex, parental obesity, parental education, monthly household income, marital status, and sleep time, in the mixed effects linear regression models (random intercept: individual).

**Table 6:** Changes in secondary outcomes of usual care and exercise groups.

Outcome Measure	Usual care Group (*n* = 44)	Exercise Group (*n* = 26)	β (95%CI) ^b^	β (95%CI) ^c^
**Body composition**				
**Waist circumference, cm**				
Baseline	93.43 ± 11.79	97.61 ± 8.59		
16-wk follow-up	93.67 ± 13.97	96.32 ± 9.68	−1.45 (−4.03 to 1.12)	−0.91 (−4.11 to 2.28)
*p*-value ^a^	0.76	0.27		
**Body fat, kg**				
Baseline	29.75 ± 9.40	32.33 ± 6.38		
16-wk follow-up	30.42 ± 9.90	31.64 ± 6.69	−1.25 (−2.22 to −0.28)	−1.12 (−2.58 to 0.34)
*p*-value ^a^	0.01	0.17		
**Body fat, %**				
Baseline	41.26 ± 4.25	41.77 ± 4.23		
16-wk follow-up	41.05 ± 4.49	40.22 ± 4.62	−1.31 (−2.07 to −0.54)	−1.52 (−2.58 to −0.45)
*p*-value ^a^	0.37	<0.001		
**Lean mass, kg**				
Baseline	40.18 ± 10.98	42.85 ± 6.41		
16-wk follow-up	41.26 ± 10.96	44.55 ± 6.04	0.76 (0.02 to 1.50)	1.20 (0.12 to 2.29)
*p*-value ^a^	<0.001	<0.001		
**Cardiometabolic risk marker**				
**SBP, mmHg**				
Baseline	123.24 ± 15.69	125.92 ± 14.99		
16-wk follow-up	123.91 ± 15.95	123.06 ± 11.07	−3.31 (−8.75 to 2.13)	−5.78 (−13.21 to 1.64)
*p*-value ^a^	0.71	0.20		
**DBP, mmHg**				
Baseline	66.67 ± 7.64	65.60 ± 6.72		
16-wk follow-up	65.59 ± 6.43	61.44 ± 9.47	−3.09 (−6.50 to 0.31)	−5.24 (−9.66 to −0.83)
*p*-value ^a^	0.27	0.02		
HOMA-IR *				
Baseline	4.17 ± 1.84	5.28 ± 2.09		
16-wk follow-up	4.43 ± 1.77	4.79 ± 1.80	−1.14 (−1.39 to 1.07)	−1.20 (−1.47 to 1.02)
*p*-value ^a^	0.36	0.24		
**HDL-C, mg/dL**				
Baseline	47.45 ± 9.51	49.54 ± 10.42		
16-wk follow-up	47.18 ± 9.97	48.42 ± 9.28	−0.50 (−3.69 to 2.69)	0.93 (−2.90 to 4.75)
*p*-value ^a^	0.81	0.30		
**LDL-C, mg/dL**				
Baseline	113.59 ± 22.62	110.15 ± 28.04		
16-wk follow-up	112.23 ± 22.44	103.88 ± 24.61	−5.12 (−12.68 to 2.43)	−4.12 (−14.96 to 6.73)
*p*-value ^a^	0.55	0.08		
TG, mg/dL *				
Baseline	98.27 ± 1.50	108.92 ± 1.63		
16-wk follow-up	97.83 ± 1.60	103.59 ± 1.60	−1.04 (−1.27 to 1.18)	−1.17 (−1.47 to 1.07)
*p*-value ^a^	0.94	0.59		
AST, U/L *				
Baseline	23.30 ± 1.53	26.23 ± 1.90		
16-wk follow-up	22.07 ± 1.56	23.73 ± 1.69	−1.05 (−1.17 to 1.07)	1.04 (−1.11 to 1.20)
*p*-value ^a^	0.13	0.06		
ALT, U/L *				
Baseline	25.30 ± 2.22	31.56 ± 2.61		
16-wk follow-up	23.05 ± 2.23	26.49 ± 2.35	−1.08 (−1.28 to 1.10)	1.01 (−1.22 to 1.25)
*p*-value ^a^	0.14	0.002		
GGT, U/L *				
Baseline	20.23 ± 1.78	23.34 ± 1.96		
16-wk follow-up	21.03 ± 1.78	20.99 ± 1.84	−1.15 (−1.27 to −1.04)	−1.08 (−1.24 to 1.05)
*p*-value ^a^	0.27	0.01		
CRP, mg/L *				
Baseline	1.44 ± 3.01	1.87 ± 2.76		
16-wk follow-up	1.53 ± 2.92	1.19 ± 2.46	−1.61 (−2.33 to −1.11)	−1.67 (−2.77 to −1.01)
*p*-value ^a^	0.60	0.02		
**Nutrition**				
**Total energy intake, kcal (*n* = 35/24)**				
Baseline	2367.2 ± 424.9	2509.8 ± 559.9		
16-wk follow-up	2022.5 ± 521.6	1965.4 ± 409.0	−244.8 (−565.5 to 75.8)	−44.9 (−429.9 to 340.0)
*p*-value ^a^	0.001	<0.001		
**Cardiorespiratory fitness and muscular strength**				
**Step test, post exam HR (*n* = 36/22)**				
Baseline	128.22 ± 22.49	123.95 ± 19.41		
16 wk follow-up	122.94 ± 18.67	120.18 ± 23.64	−7.07 (−20.95 to 6.81)	−9.52 (−26.07 to 7.03)
*p*-value ^a^	0.30	0.53		
**Arm curl test, frequency (*n* = 36/22)**				
Baseline	16.17 ± 7.20	16.86 ± 5.64		
16-wk follow-up	17.22 ± 4.32	19.36 ± 4.84	1.00 (−1.67 to 3.67)	1.47 (−2.36 to 5.29)
*p*-value ^a^	0.33	0.02		
**Wall sit test, sec (*n* = 36/22)**				
Baseline	46.12 ± 29.11	36.27 ± 23.19		
16-wk follow-up	54.92 ± 32.65	78.47 ± 40.88	34.26 (19.65 to 48.88)	50.74 (32.30 to 69.18)
*p*-value ^a^	0.07	<0.001		

Abbreviations: CI, confidence interval; wk, week; SBP, systolic blood pressure; DBP, diastolic blood pressure; HOMA-IR, homeostasis model assessment for insulin resistance; HDL-C, high-density lipoprotein cholesterol; LDL-C, low-density lipoprotein cholesterol; TG, triglyceride; AST, aspartate aminotransferase; ALT, alanine aminotransferase; GGT, gamma-glutamyl transferase; CRP, high-sensitivity C-reactive protein; HR, heart rate. HOMA-IR = (fasting plasma glucose level (in milligrams per deciliter) × fasting plasma insulin level (in microunits per milliliter))/405. Data are expressed as mean ± standard deviation unless otherwise indicated. * Geometric mean ± standard deviation; ^a^ paired *t*-test between baseline data and 16-wk follow-up data. ^b^ group × time interaction effects adjusted for age and sex in the mixed effects linear regression models (random intercept: individual). ^c^ group × time interaction effects adjusted for age, sex, parental obesity, parental education, monthly household income, marital status, and sleep time in the mixed effects linear regression models (random intercept: individual).

## References

[1] Ogden C.L., Carroll M.D., Lawman H.G., Fryar C.D., Kruszon-Moran D., Kit B.K., Flegal K.M. (2016). Trends in Obesity Prevalence Among Children and Adolescents in the United States, 1988–1994 Through 2013–2014. JAMA.

[2] Skinner A.C., Skelton J.A. (2014). Prevalence and trends in obesity and severe obesity among children in the United States, 1999–2012. JAMA Pediatr..

[3] Ng M., Fleming T., Robinson M., Thomson B., Graetz N., Margono C., Mullany E.C., Biryukov S., Abbafati C., Abera S.F. (2014). Global, regional, and national prevalence of overweight and obesity in children and adults during 1980–2013: A systematic analysis for the Global Burden of Disease Study 2013. Lancet.

[4] Whitaker R.C., Wright J.A., Pepe M.S., Seidel K.D., Dietz W.H. (1997). Predicting obesity in young adulthood from childhood and parental obesity. N. Engl. J. Med..

[5] Sachdev H.P., Osmond C., Fall C.H., Lakshmy R., Ramji S., Dey Biswas S.K., Prabhakaran D., Tandon N., Reddy K.S., Barker D.J. (2009). Predicting adult metabolic syndrome from childhood body mass index: Follow-up of the New Delhi birth cohort. Arch. Dis. Child..

[6] Juonala M., Magnussen C.G., Berenson G.S., Venn A., Burns T.L., Sabin M.A., Srinivasan S.R., Daniels S.R., Davis P.H., Chen W. (2011). Childhood adiposity, adult adiposity, and cardiovascular risk factors. N. Engl. J. Med..

[7] Weiss R., Dziura J., Burgert T.S., Tamborlane W.V., Taksali S.E., Yeckel C.W., Allen K., Lopes M., Savoye M., Morrison J. (2004). Obesity and the metabolic syndrome in children and adolescents. N. Engl. J. Med..

[8] Kelly A.S., Barlow S.E., Rao G., Inge T.H., Hayman L.L., Steinberger J., Urbina E.M., Ewing L.J., Daniels S.R. (2013). American Heart Association Atherosclerosis, Hypertension, and Obesity in the Young Committee of the Council on Cardiovascular Disease in the Young, Council on Nutrition, Physical Activity and Metabolism, and Council on Clinical Cardiology. Severe obesity in children and adolescents: Identification, associated health risks, and treatment approaches: A scientific statement from the American Heart Association. Circulation.

[9] Student Health Policy Division (2017). Announcement of Sample Results of the 2016 Student Health Examination. Ministry of Education. https://www.moe.go.kr/boardCnts/fileDown.do?m=0503&s=moe&fileSeq=2524cb8df965e5bc297eae34d7289ecf..

[10] Calcaterra V., Klersy C., Muratori T., Telli S., Caramagna C., Scaglia F., Cisternino M., Larizza D. (2008). Prevalence of metabolic syndrome (MS) in children and adolescents with varying degrees of obesity. Clin. Endocrinol..

[11] Rank M., Siegrist M., Wilks D.C., Langhof H., Wolfarth B., Haller B., Koenig W., Halle M. (2013). The cardio-metabolic risk of moderate and severe obesity in children and adolescents. J. Pediatr..

[12] Lo J.C., Chandra M., Sinaiko A., Daniels S.R., Prineas R.J., Maring B., Parker E.D., Sherwood N.E., Daley M.F., Kharbanda E.O. (2014). Severe obesity in children: Prevalence, persistence and relation to hypertension. Int. J. Pediatr. Endocrinol..

[13] Freedman D.S., Mei Z., Srinivasan S.R., Berenson G.S., Dietz W.H. (2007). Cardiovascular risk factors and excess adiposity among overweight children and adolescents: The Bogalusa Heart Study. J. Pediatr..

[14] O’Connor E.A., Evans C.V., Burda B.U., Walsh E.S., Eder M., Lozano P. (2017). Screening for Obesity and Intervention for Weight Management in Children and Adolescents: Evidence Report and Systematic Review for the US Preventive Services Task Force. JAMA.

[15] Zolotarjova J., Ten Velde G., Vreugdenhil A.C.E. (2018). Effects of multidisciplinary interventions on weight loss and health outcomes in children and adolescents with morbid obesity. Obes. Rev..

[16] Anderson Y.C., Wynter L.E., Grant C.C., Cave T.L., Derraik J.G.B., Cutfield W.S., Hofman P.L. (2017). A Novel Home-Based Intervention for Child and Adolescent Obesity: The Results of the Whānau Pakari Randomized Controlled Trial. Obesity.

[17] Rijks J.M., Plat J., Mensink R.P., Dorenbos E., Buurman W.A., Vreugdenhil A.C. (2015). Children with Morbid Obesity Benefit Equally as Children with Overweight and Obesity From an Ongoing Care Program. J. Clin. Endocrinol. Metab..

[18] Van der Baan-Slootweg O., Benninga M.A., Beelen A., van der Palen J., Tamminga-Smeulders C., Tijssen J.G., van Aalderen W.M. (2014). Inpatient treatment of children and adolescents with severe obesity in the Netherlands: A randomized clinical trial. JAMA Pediatr..

[19] Knöpfli B.H., Radtke T., Lehmann M., Schätzle B., Eisenblätter J., Gachnang A., Wiederkehr P., Hammer J., Brooks-Wildhaber J. (2008). Effects of a multidisciplinary inpatient intervention on body composition, aerobic fitness, and quality of life in severely obese girls and boys. J. Adolesc. Health.

[20] Hoedjes M., Makkes S., Halberstadt J., Noordam H., Renders C.M., Bosmans J.E., van der Baan-Slootweg O.H., Seidell J.C. (2018). Health-Related Quality of Life in Children and Adolescents with Severe Obesity after Intensive Lifestyle Treatment and at 1-Year Follow-Up. Obes. Facts.

[21] Moon J.S., Lee S.Y., Nam C.M., Choi J.M., Choe B.K., Seo J.W., Oh K., Jang M.J., Hwang S.S., Yoo M.H. (2008). 2007 Korean National Growth Charts: Review of developmental process and an outlook. Korean J. Pediatr..

[22] Styne D.M., Arslanian S.A., Connor E.L., Farooqi I.S., Murad M.H., Silverstein J.H., Yanovski J.A. (2017). Pediatric Obesity-Assessment, Treatment, and Prevention: An Endocrine Society Clinical Practice Guideline. J. Clin. Endocrinol. Metab..

[23] Kalarchian M.A., Levine M.D., Arslanian S.A., Ewing L.J., Houck P.R., Cheng Y., Ringham R.M., Sheets C.A., Marcus M.D. (2009). Family-based treatment of severe pediatric obesity: Randomized, controlled trial. Pediatrics.

[24] Lazzer S., Boirie Y., Poissonnier C., Petit I., Duché P., Taillardat M., Meyer M., Vermorel M. (2005). Longitudinal changes in activity patterns, physical capacities, energy expenditure, and body composition in severely obese adolescents during a multidisciplinary weight-reduction program. Int. J. Obes..

[25] Cho S.C., Lee Y.S. (1990). Development of the Korean Form of the Kovacs’ Children’s Depression Inventory. J. Korean Neuropsychiatr. Assoc..

[26] Knight D., Hensley V.R., Waters B. (1988). Validation of the Children’s Depression Scale and the Children’s Depression Inventory in a prepubertal sample. J. Child. Psychol. Psychiatry.

[27] Carey M.P., Faulstich M.E., Gresham F.M., Ruggiero L., Enyart P. (1987). Children’s Depression Inventory: Construct and discriminant validity across clinical and nonreferred (control) populations. J. Consult Clin. Psychol.

[28] Bull F.C., Maslin T.S., Armstrong T. (2009). Global physical activity questionnaire (GPAQ): Nine country reliability and validity study. J. Phys. Act. Health.

[29] Johnston C.A., Tyler C., Palcic J.L., Stansberry S.A., Gallagher M.R., Foreyt J.P. (2011). Smaller weight changes in standardized body mass index in response to treatment as weight classification increases. J. Pediatr..

[30] Deforche B., De Bourdeaudhuij I., Debode P., Vinaimont F., Hills A.P., Verstraete S., Bouckaert J. (2003). Changes in fat mass, fat-free mass and aerobic fitness in severely obese children and adolescents following a residential treatment programme. Eur. J. Pediatr..

[31] Ravussin E., Burnand B., Schutz Y., Jéquier E. (1985). Energy expenditure before and during energy restriction in obese patients. Am. J. Clin. Nutr..

[32] Zwiauer K.F., Mueller T., Widhalm K. (1992). Resting metabolic rate in obese children before, during and after weight loss. Int. J. Obes. Relat. Metab. Disord..

[33] Tikkanen E., Gustafsson S., Ingelsson E. (2018). Associations of Fitness, Physical Activity, Strength, and Genetic Risk with Cardiovascular Disease: Longitudinal Analyses in the UK Biobank Study. Circulation.

[34] Díez-Fernández A., Martínez-Vizcaíno V., Torres-Costoso A., Cañete García-Prieto J., Franquelo-Morales P., Sánchez-López M. (2018). Strength and cardiometabolic risk in young adults: The mediator role of aerobic fitness and waist circumference. Scand. J. Med. Sci. Sports.

[35] Shiroma E.J., Cook N.R., Manson J.E., Moorthy M.V., Buring J.E., Rimm E.B., Lee I.M. (2017). Strength Training and the Risk of Type 2 Diabetes and Cardiovascular Disease. Med. Sci. Sports Exerc..

[36] Åberg N.D., Kuhn H.G., Nyberg J., Waern M., Friberg P., Svensson J., Torén K., Rosengren A., Åberg M.A., Nilsson M. (2015). Influence of Cardiovascular Fitness and Muscle Strength in Early Adulthood on Long-Term Risk of Stroke in Swedish Men. Stroke.

[37] Kong A.S., Sussman A.L., Yahne C., Skipper B.J., Burge M.R., Davis S.M. (2013). School-based health center intervention improves body mass index in overweight and obese adolescents. J. Obes..

[38] Lisón J.F., Real-Montes J.M., Torró I., Arguisuelas M.D., Alvarez-Pitti J., Martínez-Gramage J., Aguilar F., Lurbe E. (2012). Exercise intervention in childhood obesity: A randomized controlled trial comparing hospital- versus home-based groups. Acad. Pediatr..

[39] Nemet D., Barkan S., Epstein Y., Friedland O., Kowen G., Eliakim A. (2005). Short- and long-term beneficial effects of a combined dietary-behavioral-physical activity intervention for the treatment of childhood obesity. Pediatrics.

[40] Arauz Boudreau A.D., Kurowski D.S., Gonzalez W.I., Dimond M.A., Oreskovic N.M. (2013). Latino families, primary care, and childhood obesity: A randomized controlled trial. Am. J. Prev. Med..

[41] Croker H., Viner R.M., Nicholls D., Haroun D., Chadwick P., Edwards C., Wells J.C., Wardle J. (2012). Family-based behavioural treatment of childhood obesity in a UK National Health Service setting: Randomized controlled trial. Int. J. Obes..

[42] Savoye M., Shaw M., Dziura J., Tamborlane W.V., Rose P., Guandalini C., Goldberg-Gell R., Burgert T.S., Cali A.M., Weiss R. (2007). Effects of a weight management program on body composition and metabolic parameters in overweight children: A randomized controlled trial. JAMA.

[43] Levine M.D., Ringham R.M., Kalarchian M.A., Wisniewski L., Marcus M.D. (2001). Is family-based behavioral weight control appropriate for severe pediatric obesity?. Int. J. Eat. Disord..

